# Editorial: History, advantages, complications, and limits of minimally invasive urologic pelvic surgery

**DOI:** 10.3389/fsurg.2023.1260951

**Published:** 2023-11-21

**Authors:** Ettore Mearini, Luigi Schips, Philippe Sèbe

**Affiliations:** ^1^ Department of Medicine, Urology Clinic, University of Perugia, Perugia, Italy; ^2^ Department of Medical, Oral and Biotechnological Sciences, School of Medicine and Health Sciences, Urology, G. D'Annunzio University of Chieti-Pescara, Chieti, Italy

**Keywords:** minimally invasive surgeries (MIS), robotic surgery, complications, surgical limits, laparoscopy, complications classification system

**Editorial on the Research Topic** History, advantages, complications, and limits of minimally invasive urologic pelvic surgery

Since its introduction to the medical field, minimally invasive surgery (MIS) has been a topic of debate due to concerns about safety and efficacy across various specialties.

The first laparoscopic series of cases was described by Jacobaeus in 1910. However, it was only in 1924 that the use of carbon dioxide instead of air was proposed by Zollikofer. Initially, laparoscopy was confined to the gynecological field, but it was later adopted by urology in 1976, when Cortesi used it for undescended testis. However, early experiences were fraught with difficulties, which included long operative times, surgical failures, and a high rate of complications (1, 2). In laparoscopic procedures, many of the latter were due to surgeons being unfamiliar with the technique (2, 3). However, by the 1990s, more surgeons had completed the learning curve, which led to a wider adoption of laparoscopy for many procedures (4, 5). Its advantages include a magnification of the surgical field, faster postoperative recovery, and better cosmetic results (5). During the “golden age” of laparoscopy, the development of the first robotic systems in medicine began. The necessity for remote surgeries due to global wars and the dream of men on Mars led to the development of the first master-slave robotic system in the 1990s (6). The evolution continued with the creation of an endoscopic camera manipulator that could be controlled by the surgeon's voice commands. This marked the first development of a system capable of replicating the movements of the surgeon's arm, called the ZEUS system by Computer Motion. The latter merged with Intuitive Surgical®, leading to the birth of the widely known DaVinci® robotic system (6). Further development resulted in the creation of additional robotic systems with unique characteristics, as highlighted by Dong et al. in their prospective study of retroperitoneal partial adrenalectomy (7). These robotic systems have comparable outcomes to the DaVinci® robotic system.

The widespread adoption of robotic systems in surgery can be attributed to the experience gained by many surgeons with laparoscopy and the additional benefits brought by this technique. These benefits include improved precision of movements, better surgical dissection, and enhanced bleeding control. The advantages of MIS have transformed the way surgical techniques are learned. The magnification of the surgical field has led to a redefinition of surgical boundaries, resulting in a smaller visual space and limited control of surrounding structures. The magnification and precision of movements have shifted the surgical focus from a tactile to a visual approach, leading to longer operative times but also reducing surgical and functional complications. All of the above, along with the implementation of protocols for enhanced recovery after surgery, has led to a better understanding of this type of high-cost surgery, as emphasized by Lei et al. in their contribution to this Research Topic (8).

Unfortunately, minimally invasive surgery is not always applicable, with its major limitations becoming apparent in cases of advanced disease (9–12). In such situations, complications can arise during surgery, making it even more important for surgeons and patients to make informed decisions together. The Clavien-Dindo classification system has standardized the reporting of complications since its initial implementation and has been updated with the latest EAU intraoperative adverse incident classification (13). Standardizing surgical complications and functional sequelae helps to reduce their impact, improve knowledge, and enhance treatment outcomes. However, the classification systems used in research lack a shared management approach to standardize results and lack basic tools to help patients better understand complications. The classification system enables healthcare providers to explain the possible complications associated with surgeries such as robot-assisted radical prostatectomy in advanced disease or cystectomy, as described by Cochetti et al. and Paladini et al. The Clavien-Dindo classification aims to categorize possible complications for all surgical interventions, but specific complications must be identified for each procedure (14–17).

The limits and complications of MIS compared to the open surgical technique were widely discussed when it was first introduced. However, after several years, MIS gained wider adoption, with many highly experienced open surgeons performing it. With the emergence of new technologies and technical skills in the urological field, many new urologists now have extensive MIS experience but limited experience with open surgery. Despite more than 20 years of MIS, its limitations, complications, and relative management techniques are still evolving. Therefore, it is essential to investigate the issue of limitations and complications to prevent them and make informed decisions about their management.

This research topic emphasizes the importance of applying complication classification systems to report unexpected events to improve knowledge and evidence of urological complications, with the ultimate goal of developing guidelines for complication management.
